# Care Partner Perspectives on Hypertension Management in Alzheimer Disease and Related Dementias in an Online Forum: Qualitative Thematic Analysis

**DOI:** 10.2196/85818

**Published:** 2026-07-28

**Authors:** Jisook Ko, Kylie Meyer, Jing Wang

**Affiliations:** 1 College of Nursing, University of Utah, 10 South 2000 East, Salt Lake City, UT, 84112, United States, 1 512-665-8109; 2 School of Nursing, Case Western Reserve University, Cleveland, OH, United States; 3 Florida State University, Tallahassee, FL, United States

**Keywords:** Alzheimer disease, dementia, care partner, hypertension, online health communities

## Abstract

**Background:**

Family care partners manage care for individuals with Alzheimer disease and related dementias (ADRD), including hypertension management crucial for preventing cognitive decline. Nurses frequently interact with these caregiver-patient dyads but have limited evidence about caregivers’ real-world experiences of managing this comorbidity. Understanding these experiences is essential for developing nurse-led interventions that support both patients and caregivers.

**Objective:**

This study aimed to explore ADRD care partners’ perceptions, experiences, and challenges related to hypertension management. A qualitative thematic analysis of care partner posts from an online forum focused on ADRD was conducted.

**Methods:**

We analyzed 300 posts from ALZConnected, collected using the keywords “hypertension” and “blood pressure.” Two independent researchers used reflexive thematic analysis, following the Braun and Clarke framework until data saturation was achieved.

**Results:**

Among posts from predominantly family care partners (10,427/11,092, 94%, mostly adult children), 7 themes emerged: Pharmacotherapy (155/300, 51.7%), addressing medication efficacy, polypharmacy, and side effects; Patient Health Care Implementation Strategies (n=125, 41.7%), encompassing care coordination and provider interactions; Comorbid Care Management (n=136, 45.3%), highlighting caregiving demands, behavioral symptoms of ADRD, and care coordination challenges as barriers to hypertension control; Hypertension Management and Outcomes (n=71, 23.7%), including hypertensive crises; Managing Behavioral Symptoms (n=38, 12.7%), particularly care resistance; Peer Medical Advice Exchange (n=9, 3%); and Caregiver Burden and Self-Care for Hypertension (n=25, 8.3%), revealing care partners’ own hypertension attributed to caregiving stress.

**Conclusions:**

Managing comorbid hypertension in ADRD presents challenges spanning medication management, behavioral symptoms, and health care coordination. Caregivers reported developing hypertension themselves, highlighting bidirectional health impacts. Health care providers should implement dyadic interventions addressing both patient hypertension management and caregiver health within the care partnership.

## Introduction

### Background

Alzheimer disease and related dementias (ADRD) affect approximately 7 million Americans aged 65 years and older, projected to reach 13.8 million by 2050 [1,2]. While antiamyloid therapies represent a significant advance as disease-modifying treatments, their clinical impact and accessibility remain subjects of ongoing debate; thus, prevention continues to focus on managing chronic conditions, particularly hypertension, which contributes to cerebrovascular disease, mixed pathology, and potential acceleration of cognitive decline [3-6]. Hypertension serves both as a vascular risk factor for ADRD development and as a highly prevalent comorbidity, with 83% of patients with ADRD experiencing hypertension [7]. Midlife hypertension shows consistent associations with later cognitive impairment and dementia [4,8].

Family care partners provide the majority of care for persons living with ADRD, yet they typically lack formal training in managing complex health needs such as hypertension [9,10]. This gap is particularly concerning because hypertension management encompasses multiple demanding tasks: medication administration, dietary oversight, exercise encouragement, and blood pressure (BP) monitoring, all requiring knowledge and skills that care partners may not possess.

Nearly half of caregivers providing complex care, regardless of care recipient condition, worry about making mistakes, suggesting low self-efficacy [10]. Research demonstrates that low self-efficacy when managing complex tasks, such as hypertension regimens, is associated with adverse caregiver health outcomes, including depression [11]. Behavioral and psychological symptoms of dementia (BPSD), such as refusal of care, can interfere with care delivery and exacerbate caregiver burden [12-14]. These patterns are consistent with predictions from the stress and coping model [15,16], which posits that caregiver outcomes depend on the dynamic interplay between primary stressors (care demands), secondary stressors (role strain, competing responsibilities), appraisal processes (perceived burden and self-efficacy), and coping resources (social support, problem-solving strategies). Pearlin et al [16] adapted this framework specifically for dementia caregiving, emphasizing how chronic care demands can cascade into secondary stress proliferation, affecting caregiver health and well-being. Within this framework, managing hypertension in ADRD represents a significant primary stressor, one that, when compounded by low caregiver self-efficacy and BPSD, may contribute to the broader pattern of caregiver health decline documented in the literature.

Within this framework, managing hypertension in ADRD represents a significant primary stressor. Nearly half of caregivers providing complex care, regardless of care recipient condition, worry about making mistakes, suggesting low self-efficacy [10]. According to the stress and coping model, low self-efficacy when providing complex care, such as hypertension management, among ADRD caregivers contributes to adverse caregiver health outcomes, such as depression [15]. BPSD, such as refusal of care, can interfere with care delivery and exacerbate caregiver burden [11,12,17].

Care partners performing health management tasks report reduced social participation [13], while those with greater social support experience lower burden [14,18]. Online support communities provide caregivers with accessible platforms for social support, including information seeking, emotional support, and shared decision-making [19-21]. A meta-analysis showed positive impacts of internet-based group support on caregiver social support and self-efficacy [22]. Specifically, this meta-analysis synthesized findings from internet-based group support programs—structured, moderated online interventions designed to deliver peer connection, psychoeducation, and coping skills to dementia care partners via videoconferencing or asynchronous message boards—and found improvements in perceived social support, self-efficacy, and reductions in burden across studies. A scoping review of online support forums further identified ALZConnected [23]—the forum examined in this study—as one of the largest and most active peer-to-peer support communities for dementia care partners, with users exchanging practical advice, emotional support, and shared coping strategies asynchronously [24]. Despite these benefits, peer advice exchanged in unmoderated or lightly moderated forums may include medically inaccurate information, highlighting the need for nurses to understand what care partners encounter and discuss in these spaces.

The psychosocial burdens described above do not occur in isolation from physical health. A growing body of evidence demonstrates that the chronic stress of ADRD caregiving has direct cardiovascular consequences. Older adults who provide care to a spouse or family member with dementia have significantly elevated rates of hypertension compared with noncaregiving peers [25,26]. Longitudinal studies indicate that caregiving stress accelerates the development of hypertension and increases the risk of incident cardiovascular disease, even after adjusting for age and baseline health status [16,26]. Two intersecting pathways explain this elevated risk. First, caregivers may develop hypertension de novo as a physiological response to chronic stress, through sustained activation of the hypothalamic-pituitary-adrenal axis and sympathetic nervous system. Second, caregivers who had pre-existing hypertension often experience worsening BP control as caregiving demands displace time and energy for their own health behaviors, including medication adherence, dietary management, and medical appointments [25]. Despite this dual burden—managing their care recipient’s hypertension while their own BP goes unmanaged—caregiver cardiovascular health is rarely addressed in clinical encounters focused on the person with ADRD. Nurses are well positioned to bridge this gap, yet they need a clearer understanding of what care partners experience in managing hypertension across the dyad to design effective, targeted interventions.

### Objectives

To our knowledge, no study has analyzed online community health data to explore the dual challenges of providing hypertension management to persons living with ADRD while managing caregivers’ own hypertension-related health needs. This study examined 2 interconnected aspects: (1) caregivers’ perceptions and challenges in managing hypertension for individuals with ADRD and hypertension and (2) caregivers’ own hypertension management challenges contributing to their chronic disease burden. Findings will inform nursing interventions supporting caregiver-patient dyads.

## Methods

### Study Design

This exploratory qualitative study used reflexive thematic analysis, following the Braun and Clarke (2006) 6-phase framework [27] to explore ADRD caregivers’ experiences with hypertension management. This inductive approach allowed themes to emerge from the data rather than imposing pre-existing theoretical categories.

### Study Setting and Data Source

Data were extracted from ALZConnected [23], a moderated online community platform established by the Alzheimer’s Association. The forum provides message boards where care partners and persons across the ADRD continuum—including those with mild cognitive impairment and established dementia—discuss various topics related to dementia care. This forum represents the heterogeneity of ADRD subtypes rather than reflecting a particular disease experience. While users must register to post content, all posts are publicly accessible without authentication. At the time of data extraction, 11,092 registered users contributed to the forum, with 94% (n=10,427) self-identifying as family caregivers (n=7,543, 68% adult children; n=1,331, 12% partners/spouses; n=776, 7% other relatives; n=776, 7% grandchildren/siblings);2% (n=222) as professional caregivers; and <1% (n=111) as persons with dementia; and 3% (n=333) did not specify a role.

### Data Collection

Posts were extracted via web scraping between May 21 and 28, 2019. We used the search terms “hypertension” OR “ blood pressure” to identify potentially relevant posts, yielding an initial set of 1357 results. Posts were subsequently reviewed to confirm that each referenced both a care recipient with ADRD and the management of hypertension or blood pressure; posts discussing only one of these topics were excluded. From this filtered dataset, we systematically analyzed posts until reaching data saturation at 300 posts.

### Ethical Considerations

This study was determined to be exempt from full ethics review by the institutional review board at the University of Texas Health Science Center at Houston (HSC20200784N), as it used deidentified, publicly available online forum data. All forum posts are publicly accessible without authentication, and usernames are pseudonyms; no personally identifiable information was collected or retained.

### Data Analyses

Analysis followed a systematic 6-phase process:

- *Phase 1—Familiarization*: 2 trained individuals independently read all extracted posts (n=1357) to gain data immersion.
- *Phase 2—Initial coding*: using manual coding, researchers independently applied open coding to identify meaningful units within posts. An initial codebook containing 14 codes was developed after coding the first 50 posts.
- *Phase 3—Code refinement*: researchers independently coded an additional 100 posts, refining code definitions and identifying 9 additional codes, resulting in 23 total codes.
- *Phase 4—Theme development*: researchers met to review all codes and organize them into potential themes through iterative discussion and consensus building.
- *Phase 5—Theme refinement*: continuing independent coding, researchers achieved saturation at 300 posts, defined as the point at which no new codes emerged across 3 consecutive 25-post batches. All 300 posts were then reviewed to ensure thematic coherence and internal consistency.
- *Phase 6—Theme finalization*: the coding team, including a senior qualitative researcher (with more than 10 years’ experience), met to finalize theme names, definitions, boundaries, and relationships. Disagreements were resolved through discussion until consensus was reached.

Each post could be assigned multiple codes reflecting different dimensions of hypertension management experience. Final themes were organized hierarchically with primary themes and constituent subcodes (Table 1).

**Table 1.** Full list of all codes generated from the data.

Code name (number of posts)	Description
Theme 1: Pharmacotherapy (155/300, 51.7%)	
Medication in Hypertension Management (n=94)	Management of hypertension with medication(s). Includes both proper and poor management of hypertension with medication.
Medication Management (n=26)	Caregiver opinion or evaluation of the list of medications a loved one was taking or was prescribed. Includes caregivers having added or removed medications with or without physician approval.
Hypertension Medication Side Effects (n=24)	Side effects presumed from a hypertension medication, such as fainting, dizziness, and falls.
Medication Administration (n=11)	Logistics of medication administration, including ways that caregivers have gotten care recipients to take their medications, such as through the use of pill dispensers or crushing medication into food.
Theme 2: Patient Health Care Implementation Strategies (125/300, 41.7%)	
Care Coordination (n=57)	Coordination of care by the caregiver for an individual with ADRD^a^. Includes topics such as medicolegal issues, finding housing care, and decisions about hiring home care services to ease caregiver burden.
Interaction With Health Care Team (n=30)	Caregiver opinions on their interactions with, or decisions made by, medical personnel (any medical professional or hired caregivers) in charge of or familiar with their loved one’s medical needs and care.
Responsibility (n=20)	Where the hypertension care management responsibility falls in terms of who is responsible and how much they are responsible for, such as if the individual with ADRD manages it themselves or if this is put on a caregiver.
Blood Pressure Evaluation (n=10)	Evaluating a loved one’s blood pressure by either a physician or themselves as the caregiver. Includes confirmation or reevaluation of a previous hypertension diagnosis or taking a blood pressure reading and evaluating what to do.
Palliative Care Versus Health Management (n=8)	Decision of entering palliative care and how to address hypertension in this stage, if at all. Caregivers also discuss whether or not to continue hypertension medications when in palliative care or hospice.
Theme 3: Comorbid Care Management (136/300, 45.3%)	
Multiple Chronic Conditions Management (n=112)	Having a hypertension diagnosis along with another definitive disease diagnosis, including but not limited to ADRD, physical illness, and mental illness.
Caregiver’s Theories About ADRD and Hypertension Relationships (n=13)	Speculation or confirmation of the link between ADRD and hypertension as perceived by the caregiver, author of the post. Discussed either as passing speculation or with supporting articles and reasoning.
ADRD as a Barrier to Hypertension Management (n=11)	How an ADRD diagnosis or its symptoms, such as forgetfulness or anosognosia, affect the care management of hypertension.
Theme 4: Hypertension Management and Outcomes (71/300, 23.7%)	
Hypertensive Episodes (n=23)	Particular occasions, time points, isolated situations, or environments that evoked a temporary elevation in blood pressure. A chronic hypertension diagnosis was not necessary for an episode to occur.
Consequences of Noncompliance (n=12)	Effects of not complying with proper hypertension care, or the consequences of poor or noncompliant hypertension management, such as further disease or adverse health events.
Possible Hypertension (n=11)	Appearance or mention of signs and symptoms of hypertension without simultaneous mention of a concrete diagnosis. Assumed to have hypertension but is not confirmed through the post.
Hypertension as a Side Effect (n=10)	Treatment, medication, or underlying comorbidities that may elicit hypertension as a side effect, such as certain oils or over-the-counter therapies.
Hypertension Exacerbation (n=8)	Situations or stressors, usually chronic, that result in the exacerbation of preexisting hypertension. Does not relate to posts for an undiagnosed person having a hypertensive episode.
Hypertension to Normotension/Hypotension (n=7)	A person going from explicitly diagnosed hypertension to a state of normal blood pressure or even hypotension. Causes for this can include ADRD progression or as a result of or side effect of medications given.
Theme 5: Managing Behavioral Symptoms (38/300, 12.7%)	
Resistance to Care (n=31)	ADRD individual being resistant to care, creating complications for the caregiver when trying to provide care to their loved one. Anosognosia or disease progression leads to ADRD individuals resisting medical care, medications, appointments, and general management of their care.
Strategies for Compliance (n=7)	Strategies that caregivers employ, create, or discuss in an attempt to get their loved one to comply with care. Strategies often include “fiblets” or white lies to achieve compliance or cooperation in tasks such as visiting the doctor.
Theme 6: Peer Medical Advice Exchange in Online Forum (9/300, 3%)	
Unqualified Medical Advice in a Forum (n=9)	Forum user or caregiver who either takes medical advice from another user on the forum or gives their own medical advice within the forum with intentions to guide or assist another caregiver in the decision-making process.
Theme 7: Caregiver Burden and Self-Care for Hypertension (25/300, 8.3%)	
Caregivers With Hypertension (n=25)	Caregivers discuss their own hypertension, whether this is diagnosed, chronic, acute, spontaneous, or a concern for development in the future. Many caregivers attribute high blood pressure, whether diagnosed or situational, to the stress and demands of the caregiving role.

a ADRD: Alzheimer disease and related dementias.

## Results

### Overview of Thematic Analysis

Through reflexive thematic analysis of 300 posts from the ALZConnected online community forum, we identified 7 major themes reflecting the multifaceted nature of hypertension management in persons with ADRD. These themes captured both care partners’ experiences of managing their care recipients’ hypertension and their own hypertension-related challenges. Table 1 presents the themes, their constituent codes, and frequency distributions. In the narrative that follows, codes within each theme are discussed according to three criteria: (1) frequency—codes appearing in a greater number of posts are given priority to reflect the breadth of care partner experience, (2) prominence—codes that were most central to the theme’s conceptual meaning receive fuller treatment regardless of raw count, and (3) richness of illustrative quotations—codes supported by particularly vivid or representative posts are highlighted to preserve the depth of care partner voice. Where codes were closely related in content or addressed the same underlying experience, they were analytically subordinated under a higher-order code and discussed together to avoid redundancy; this is noted explicitly within each theme narrative below.

### Theme 1: Pharmacotherapy (n=155, 51.7%)

#### Overview

This theme captured medication-related aspects of hypertension management, including efficacy concerns, polypharmacy challenges, side effects, and administration difficulties. Four codes comprised this theme: Medication in Hypertension Management (n=94), Medication Management (n=26), Hypertension Medication Side Effects (n=24), and Medication Administration (n=11).

#### Medication in Hypertension Management

Caregivers frequently discussed inadequate BP control despite medication use, expressing frustration with treatment failures:

*She (care recipient) has had very high blood pressure for about 10 years that medication is not controlling as well as it should*.

These posts revealed caregivers’ concern about persistent hypertension despite adherence to prescribed regimens.

#### Medication Management

Caregivers independently evaluated and modified medication regimens, sometimes without provider consultation. Caregivers expressed skepticism about polypharmacy, perceiving that providers prescribed medications to treat side effects of other medications rather than addressing root causes:

*My mom was on ten plus meds when she was diagnosed with ALZ/Dementia. I went through them and discovered her doctor was just prescribing meds, many of which were dealing with symptoms of previous meds....Anyway, I had her go cold turkey on the meds [so] we could ease her off of the meds [and] that needed to be done slowly (i.e. Prozac)*.

This quote illustrates care partners’ autonomous decision-making regarding complex medication regimens, including the abrupt discontinuation of some medications. Notably, the post does not indicate whether medical supervision was involved, and such decisions may reflect distrust of or limited access to provider guidance rather than the absence of oversight.

### Theme 2: Patient Health Care Implementation Strategies (n=125, 41.7%)

#### Overview

This theme encompassed caregivers’ roles in coordinating health care, navigating the health care system, and managing relationships with providers. Five codes comprised this theme: Care Coordination (n=57), Interaction With Health Care Team (n=30), Responsibility (n=20), Blood Pressure Evaluation (n=10), and Palliative Care Versus Health Management (n=8).

#### Care Coordination Complexity

Caregivers described extensive coordination responsibilities spanning medicolegal arrangements (power of attorney, advance directives), specialist appointments, home care services, and housing transitions. Posts revealed the logistical burden of orchestrating multidisciplinary care:

*Seven months ago my 66 yo SIL (sister in-law) came to a new state to live with my husband and me after a hospitalization due to a perfect storm of heavy drinking/high blood pressure/dementia. We established her with a new primary care, neurologist and psychiatrist*.

This example demonstrates the extensive coordination required following acute events, including establishing care in new locations and navigating insurance coverage for multiple services.

#### BP Evaluation

Caregivers discussed conducting home BP monitoring and interpreting readings, often without clear guidance on appropriate parameters or when to seek medical intervention:

*I’ve been checking Dad’s blood pressure at home like the doctor suggested, but I’m confused about what’s actually concerning. This morning it was 158/92, this afternoon 142/88. His doctor said to ‘keep an eye on it’ but didn’t tell me what numbers mean I should call. Is 158/92 an emergency? Do I wait and see if it goes down? I’m keeping a log but honestly don’t know what I’m looking for*.

### Theme 3: Comorbid Care Management (n=136, 45.3%)

#### Overview

This theme reflected caregivers’ understanding of hypertension as one condition among multiple comorbidities and their perception that ADRD complicated hypertension management. Three codes comprised this theme: Multiple Chronic Conditions Management (n=112), Caregivers’ Theories About ADRD and Hypertension Relationships (n=13), and ADRD as a Barrier to Hypertension Management (n=11).

#### Multiple Chronic Conditions Management

Caregivers described managing hypertension alongside numerous other conditions, revealing the complexity of care for medically complex older adults:

*My dad is very ‘weep’, just diagnosed two weeks ago after countless falls and cognitive issues. He has other issues, CPAP machine use, 30 pounds overweight, glaucoma and meds taking for high blood pressure. He also suffers from anxiety*

This quote exemplifies the clustering of chronic conditions (ADRD, hypertension, obesity, glaucoma, sleep apnea, and anxiety) requiring simultaneous management.

#### Caregivers' Theories About ADRD and Hypertension Relationships

Some caregivers speculated about causal relationships between hypertension and ADRD development, drawing connections between poor vascular health and cognitive decline:

*Mom has had high blood pressure & cholesterol issues for most of her life so at 79 perhaps this is the result. Lack of blood oxygen to the brain, etc*.

#### ADRD as a Barrier to Hypertension Management

Caregivers identified cognitive impairment as directly interfering with hypertension self-management by the care recipient with ADRD, including tasks such as remembering medications, understanding dietary restrictions, and recognizing symptoms requiring medical attention:

*The hardest part is the medication schedule. Mom takes her blood pressure pill in the morning and another one at night, but she forgets constantly. I’ll find her pills still in the container at bedtime, or she’ll ask me three times in one day if she already took them. Last week, she took her morning dose twice because she couldn’t remember taking it the first time*.

### Theme 4: Hypertension Management and Outcomes (n=71, 23.7%)

#### Overview

This theme captured acute hypertensive situations, consequences of poor control, and care partners’ observations of BP fluctuations. Six codes comprised this theme: Hypertensive Episodes (n=23), Effects of Hypertension Noncompliance (n=12), Possible Hypertension (n=11), Hypertension as a Side Effect (n=10), Hypertension Exacerbation (n=8), and Hypertension to Normotension/Hypotension (n=7).

#### Acute Hypertensive Episodes and Stress Reactivity

Caregivers described observing acute BP elevations, often triggered by emotional distress or behavioral symptoms:

*Decided to check blood pressure since she is so upset and it’s off the charts. I try to calm her down and within five minutes or so blood pressure is back to normal. Afraid she is going to have a heart attack or stroke*!!

This quote reveals caregivers’ recognition of stress-BP relationships and their fear of acute cardiovascular events.

#### Consequences of Noncompliance

Caregivers connected medication nonadherence to adverse outcomes, particularly cardiovascular events and dementia progression:

*Don’t want to scare you, but my mom’s dementia got worse when she had a heart attack. Her heart attack was caused by high blood pressure because she was taking her meds in a sporadic manner.*

### Theme 5: Managing Behavioral Symptoms (n=38, 12.7%)

#### Overview

This theme addressed BPSD that complicated hypertension management. Two codes comprised this theme: Resistance to Care (n=31) and Strategies for Compliance (n=7).

#### Resistance to Care

Caregivers frequently described care recipients’ refusal of medical appointments, medications, or BP monitoring, often attributing resistance to anosognosia (lack of illness awareness):

*My mom refuses medical care also. Like your dad she could care less what anyone else is doing or what their opinion is*.

This resistance created significant management challenges, forcing caregivers to balance respecting autonomy with ensuring necessary care.

#### Strategies for Compliance

Caregivers shared strategies for circumventing resistance, including “therapeutic fibbing” or “white lies”:

*Fib, tell her it’s a new program that they are using to train young nurses and caregivers, and her insurance wants her to “volunteer...” worth a shot that worked for my mom. [I] told her the insurance company required these for training and a study, to see if blood pressure taken at home was lower than when taken at the doctor’s office*.

### Theme 6: Peer Medical Advice Exchange in Online Forum (n=9, 3%)

This small but notable theme captured instances of forum users providing medical advice or recommendations to other caregivers, often without identifying professional credentials or citing evidence sources. One code comprised this theme: Peer Medical Advice (n=9).

Caregivers offered opinions on medication efficacy, treatment decisions, and care strategies based on personal experiences:

*When I read that your Mom “came back” after only 2 days of restarting the Aricept, it just solidified my opinion that this drug may help more than the Drs even know….Its only been on the market for 12 years, which is not a SUPER long time in the pharmaceutical industry*.

### Theme 7: Caregiver Burden and Self-Care for Hypertension (n=25, 8.3%)

This theme revealed caregivers’ own hypertension experiences, often attributed to caregiving stress. One code comprised this theme: Caregivers With Hypertension (n=25).

Caregivers disclosed developing hypertension or experiencing BP elevations they directly linked to caregiving demands:

*I was so frustrated with them and the situation I was literally getting sick myself. I needed counselling myself, my blood pressure went up (now I’m on BP meds); my stomach was always in knots*.

This quote exemplifies the bidirectional perceived health impact of caregiving: care partners not only managed their care recipients’ hypertension but also attributed the development of hypertension themselves as a manifestation of chronic stress. The psychosomatic framing (“literally getting sick myself”) reflected caregivers’ understanding of stress-health connections. Few care partners discussed self-care strategies, suggesting limited attention to their own health needs relative to care recipient needs.

## Discussion

### Study Overview

This qualitative analysis of 300 posts from the ALZConnected forum revealed that informal care partners of persons with ADRD managing comorbid hypertension navigate multifaceted challenges spanning clinical decision-making, behavioral symptom management, and health care system coordination—often developing hypertension themselves as a consequence of caregiving. These findings illuminate the complex intersection of dementia caregiving and chronic disease management, highlighting both the clinical challenges care partners face and the dual role of online forums as sources of peer support and potential misinformation. Importantly, these findings should be interpreted with attention to the population they most likely represent: caregivers who are digitally engaged, motivated to seek peer support, and comfortable sharing health-related experiences in online communities. This profile suggests a relatively activated, help-seeking subset of the broader caregiving population—one that may have higher health literacy, greater social connectedness, or more access to resources than caregivers who do not engage with online forums. Clinicians and researchers should consider how these characteristics may shape the themes identified and whether caregivers who are less digitally engaged or more socially isolated face additional or distinct challenges not captured in this dataset.

### Principal Results

The findings from this analysis reflect the profound daily burdens described in existing caregiving literature while offering additional context about how those burdens manifest specifically in the management of hypertension. Posts in the forum frequently depicted care partners as primary decision-makers for complex, chronic disease management—roles for which many felt underprepared. The co-occurrence of ADRD and hypertension created particularly layered challenges, as cognitive and behavioral symptoms of dementia often complicated adherence to hypertension regimens. That some care partners also reported developing hypertension themselves further illustrates how the caregiving role can blur the boundary between caregiver and care recipient, with implications for how clinicians conceptualize and address the health of the caregiving dyad.

### Comparison With Prior Work

ADRD caregiving places heavy emotional and practical demands on family members. Care partners often spend more than 150 hours per month helping with difficult cognitive and behavioral changes [28]. Caregiving can cause long-term physical and psychological strain and create extra stress in multiple life domains, such as work and family relationships, which may lead to health issues, including hypertension [29]. Research demonstrates that caregiving stress increases the risk of developing hypertension and heart disease [25]. Caregivers experiencing high levels of stress frequently neglect their own health-promoting behaviors and self-care. Studies showed that caregiving duties, lack of energy, and breakdown of social networks lead caregivers to defer their own care [16,26]. The posts analyzed in this study suggest that some care partners may manage both their loved ones’ BP and their own, resulting in ongoing health challenges for both.

Major interrelated clinical challenges emerged from the analysis.

The theme of *Pharmacotherapy* revealed patterns of autonomous decision-making, with care partners independently modifying or discontinuing medications without consulting health care providers. For example, care partners described making unilateral decisions about BP medications. Although care partners value their loved one’s autonomy in medication management, transitions to care partners taking over medication responsibilities often occur after care partners observe medication errors [30]. Care partners in the online forum frequently reported making decisions about medication changes while facing challenges related to managing multiple medications and complex dosage regimens [31]. These accounts align with research showing that caregivers assisting with medication management are often untrained, underresourced, and unsupported, even though caregivers of persons living with ADRD are often required to manage multiple simultaneous medications and complex regimens [32]. Care partner forum comments suggest that the autonomous decision-making described likely results from several factors: distrust related to perceived overmedication, concerns about certain medications and possible drug interactions, limited understanding of drug effects, and a lack of caregiver-focused medication education during clinical encounters. These findings suggest that nurses may be well positioned to address these concerns by providing caregiver education about medication management, including how to communicate concerns about overmedication with health care providers to avoid risky medication changes.

The theme of *Patient Health Care Implementation Strategies* revealed that care partners in this sample described extensive and often uncompensated coordination work—arranging specialist appointments, navigating medicolegal decisions, managing home care transitions, and monitoring BP without clear clinical guidance on when readings warranted intervention. These findings align with a growing literature documenting that family care partners frequently assume quasi-clinical roles for which they receive little preparation or support [9,10]. The gap between what care partners were asked to do—conduct and interpret home BP monitoring, coordinate multidisciplinary care teams—and the guidance they received during clinical encounters was a recurring source of frustration and uncertainty. These accounts underscore the need for explicit caregiver education at clinical touchpoints, including clear parameters for when to act on home BP readings and how to navigate specialist referral pathways.

The theme of *Comorbid Care Management* revealed that care partners consistently described hypertension not as an isolated condition but as one element of a dense cluster of comorbidities, including obesity, sleep apnea, glaucoma, anxiety, and ADRD itself. This framing reflects the clinical reality of older adults with ADRD, who carry an average of 5 or more chronic conditions simultaneously [4]. Notably, care partners in this forum identified ADRD’s cognitive and behavioral features—memory failure, anosognosia, and care resistance—as direct impediments to hypertension self-management by the care recipient. This is an important mechanistic observation: the disease that motivates hypertension management also undermines the patient’s capacity to participate in it. Clinicians should anticipate this dynamic and explicitly shift responsibility and education toward the care partner rather than assuming the person with ADRD can self-manage.

The theme of *Managing Behavioral Symptoms* revealed that care resistance—rooted in anosognosia or disease progression—emerged as a substantial impediment to routine hypertension management, including medication administration, BP monitoring, and medical appointments. Care partners described developing workarounds such as therapeutic fibbing to achieve cooperation, reflecting the extent to which they absorbed the clinical burden of managing a patient who could not consent to or comprehend their own care. These strategies, while pragmatically effective, raise ethical questions about autonomy and dignity that care partners were navigating without professional guidance. This theme reinforces the value of behavioral symptom training for care partners and highlights a gap in current clinical practice: the management of ADRD-related care resistance is rarely addressed in the context of chronic disease management protocols.

The theme of *Caregiver Burden and Self-Care for Hypertension* revealed that some care partners shared that they developed high BP or saw their BP rise because of the stress of caregiving, even though this topic only came up in a small number of posts. One caregiver said, “I was literally getting sick myself...my BP went up (now I’m on BP meds).” This quote illustrates how caregiving can affect both the caregiver and the person receiving care. Caregivers often had to handle their loved ones’ high BP while also dealing with their own. These accounts are consistent with research suggesting that caregiving can cause ongoing physical and mental strain, leading to adverse health outcomes, including hypertension [29]. Not many care partners discussed their own health or ways to care for themselves, which may be because they focus more on their loved ones’ needs than on their own, even though many caregivers have high BP. This pattern means that nurses should consider asking caregivers about their own health during appointments with care recipients, including urging care partners to maintain their own medical appointments. Nurses and other health care providers can check for caregiver stress and high BP, discuss health risks, and encourage caregivers to prioritize their own self-care. Health care systems might consider developing programs that assist care partners in managing both the daily responsibilities of caregiving and the associated health effects, such as offering stress management, health screenings, and practical support. The Guiding an Improved Dementia Experience (GUIDE) pilot program being offered through Medicare, as well as new billing codes to address the needs of care partners, are important advancements that allow nurses to implement person- and family-centered care to support caregivers [33].

Given these substantial burdens, online support groups have emerged as vital resources where care partners seek social support to buffer against the negative health impacts of caregiving [24]. These online platforms provide accessible spaces for informational, emotional, and peer support among individuals who share similar experiences. Notably, online forums offer care partners safe spaces to disclose experiences that might be stigmatized in offline settings, such as expressions of anger or negative emotions toward the care recipient, with such disclosures typically met with understanding and reciprocal narratives from other group members [34]. According to the stress process model of caregiving, access to social support, such as that from peers, has the potential to improve an individual’s appraisal of a stressor and buffer the impact of caregiving demands [16,35]. The forum analyzed in this study functioned as precisely this type of space, where caregivers disclosed their daily struggles with others navigating similar circumstances.

Due to the heavy demands on caregivers, online support groups have become crucial places to find help and connection [24]. These forums provide caregivers with a way to support one another, especially for those who live far from others or have limited time. Research shows that caregivers with the highest needs are often the most active in these groups [34]. Peer support can help caregivers handle stress and feel less alone [35]. The forum in this study served as a venue where caregivers could discuss their daily challenges with others facing similar issues. By sharing their experiences, such as discussing ways to help loved ones take their medicine or finding comfort in each other’s stories, caregivers received both helpful advice and emotional support that they might not have otherwise. At the same time, some of the information provided on online forums may not be accurate. Nurses could have a role as facilitators within these forums to provide accurate information and debunk potentially harmful advice.

Across all 7 themes, several cross-cutting patterns emerged that reflect broader systemic challenges in dementia caregiving. Care partners consistently reported unmet informational needs—particularly regarding medication management, behavioral symptom interpretation, and care coordination—that were not adequately addressed during clinical encounters. A recurring consequence was autonomous or underinformed decision-making, in which care partners acted independently due to insufficient guidance from health care providers. Fragmented care coordination further compounded these challenges, with care partners frequently navigating multiple specialists and systems without centralized support. Together, these patterns suggest that the needs of care partners managing comorbid hypertension in persons with ADRD extend well beyond the clinical encounter and underscore the value of proactive, family-centered approaches to chronic disease management in this population.

### Limitations

These findings should be viewed in light of several limitations. First, the data come from a single online forum (ALZConnected), so they may not reflect the experiences of all caregivers, especially those who do not use online communities or who use different platforms. Caregivers who post online may differ from those who do not in terms of stress, support-seeking, or health care knowledge. Second, since the forum posts are cross-sectional, we cannot track how caregiving experiences and challenges change over time. Third, limited demographic information about forum users made it impossible to analyze differences by age, gender, relationship to the care recipient, or socioeconomic status. The forum data also lacked race and ethnicity information, limiting generalizability to minoritized communities that may face distinct caregiving burdens and health disparities; future studies should intentionally recruit diverse samples. Additionally, we were unable to verify the specific etiology of care recipients’ dementia (eg, Alzheimer disease vs vascular or mixed pathology) or other clinical details, which may influence caregiving challenges and the relevance of hypertension management. Fourth, geographic data were unavailable, limiting interpretation of findings related to access to care. Fifth, the self-reported, unverified nature of online forum posts introduces self-report and potential reporting bias. Sixth, the search strategy relied exclusively on fully spelled-out terms (“hypertension” and “BP”) and did not include common medical abbreviations such as “hypertension,” “BP,” “systolic blood pressure,” or “diastolic blood pressure.” Caregivers with greater medical familiarity or clinical backgrounds may preferentially use these abbreviations, and potentially relevant posts from more health-literate users may therefore have been excluded from the dataset. This represents an additional source of selection bias that could skew findings toward caregivers with less clinical knowledge, and future studies should test whether abbreviation-based search terms yield meaningfully different or additional posts. Finally, thematic analysis is inherently interpretive; future mixed methods studies linking qualitative themes to clinical or registry data would strengthen validation of these findings. Prospective studies with comprehensive demographic data and registry-based diagnosis verification are needed to address these limitations.

### Conclusions

This analysis of online forum posts shows that care partners for people with ADRD and high BP face many challenges, such as handling medications, managing difficult behaviors, working with the health care system, and dealing with their own health. In the current sample, care partners frequently reported making medication-related decisions independently, struggled to differentiate between transient and chronic BP elevations, and sought ways to manage care resistance associated with anosognosia. These findings highlight significant gaps in the training and support available to caregivers. Health care providers and systems should recognize that care partners are essential members of the care team, but they often lack the preparation and support they need.

Future research should investigate ways to address the challenges highlighted here, such as training programs for care partners on medication management, tools to aid in interpreting BP readings, and methods for managing care resistance in individuals with limited awareness of their illness. Long-term studies could help us understand how caregivers’ needs change as dementia progresses. Research on how professional moderators influence online support communities could help identify the most effective ways to support caregivers and prevent the spread of incorrect information. Additional studies are also needed to examine caregivers’ heart health and whether targeted programs can help prevent or manage high BP in caregivers.

By learning from the experiences shared in these online forums, health care providers and systems can better support care partners in their crucial role and attend to their own health needs. Equipping care partners with targeted education, care coordination support, and access to reliable health information represents a meaningful step toward addressing the dual burden documented in this study.

## Abbreviations

ADRD: Alzheimer disease and related dementias
BP: blood pressure
BPSD: behavioral and psychological symptoms of dementia
GUIDE: Guiding an Improved Dementia Experience
